# Zolpidem for Catatonia Refractory to Benzodiazepines in Resource-Limited Settings

**DOI:** 10.1192/bjo.2025.10730

**Published:** 2025-06-20

**Authors:** Holly Hodge, Shaikh Ullahansari, Blake Berry

**Affiliations:** 1 Texas A&M College of Medicine, Dallas, USA; 2 John Peter Smith Hospital, Fort Worth, USA

## Abstract

**Aims:** Catatonia is a neuropsychiatric syndrome characterized by a paucity of movement and speech. Benzodiazepines are the mainstay of treatment for catatonia, but a subset of patients do not respond. While electroconvulsive therapy is another treatment option for catatonia, access can be limited. This case report discusses the use of zolpidem, a hypnotic non-benzodiazepine GABA-A receptor modulator, in a patient with benzodiazepine-resistant catatonia.

**Methods:** A 19-year-old male presented to a hospital in the United States from jail custody with altered mental status. His medical history was notable for psychosis with paranoid delusions and severe catatonia.

The patient reportedly had severely reduced oral intake for the past month, with almost no intake at all in the prior eight days. His presentation, which included psychomotor retardation, mutism, and posturing, raised a high clinical suspicion for catatonia.

Initial treatment with 1 mg of lorazepam every 6 hours was ineffective, and the patient developed tachycardia, raising concern for malignant catatonia. At this time, his differential diagnosis included schizophrenia. His treatment regimen was adjusted to include olanzapine for suspected schizophrenia, memantine for catatonia treatment augmentation, and metoprolol for tachycardia. Despite this regimen, along with escalating lorazepam doses up to 7 mg three times daily, the patient remained severely catatonic. A trial of clozapine also failed to yield significant improvement.

Given the patient’s limited response to benzodiazepines, zolpidem was introduced. He showed rapid improvement in his catatonic symptoms, including markedly improved speech, oral intake, and overall participation. Zolpidem was dosed throughout the day to limit drowsiness. By day 30, the patient demonstrated substantial recovery, with minimal catatonic symptoms and improved engagement in daily activities. He was discharged home on a regimen of zolpidem, clozapine, and memantine.

**Results:** A subset of patients with catatonia fail to respond to benzodiazepines, necessitating alternative treatments. Prompt intervention is crucial due to the life-threatening nature of catatonia and its numerous complications. While electroconvulsive therapy is often effective, its availability can be limited. Zolpidem, acting through a distinct mechanism of GABA-A receptor subunit binding, may offer an effective alternative for benzodiazepine-resistant cases.

**Conclusion:** Further research into zolpidem and other alternative therapies for catatonia is warranted, especially in settings where electroconvulsive therapy is not accessible. Zolpidem’s potential as a treatment for benzodiazepine-refractory catatonia deserves further investigation.

